# Association between public space and resident outdoor activity behavior in urban areas surrounding lakes

**DOI:** 10.1038/s41598-025-28431-6

**Published:** 2025-12-29

**Authors:** Fan Liya, Lai Yuqing, Hu Zhouni, Zheng Wenhui, Zhou Tao

**Affiliations:** ¹School of Architecture and Design, Nanchang University, Nanchang, China

**Keywords:** Urban areas surrounding lakes, Behavior setting theory, Resident activity behavior, GIS spatial analysis, Place symbiosis, Public space renewal, Environmental social sciences, Psychology and behaviour

## Abstract

This study integrates Behavior Setting Theory with GIS spatial analysis to elucidate the association between public space characteristics and resident outdoor activity behavior in urban areas surrounding lakes, using Nanchang’s Qingshan Lake as a case study. Applying this integrated framework, we systematically analyzed the spatio-temporal distribution of resident activities, activity type preferences, and their interactions with the spatial environment. Employing multi-source data, we developed a dynamic “people-space” interaction model. The empirical findings led to the proposal of targeted micro-renewal strategies for public spaces surrounding lakes, focusing on five key aspects: enhancing safety and resilience, ensuring seamless connectivity, promoting ecological integration, creating narrative environments, and shaping spatial affordances. This research provides a scientific basis for improving the quality of public spaces surrounding urban lakes and resident well-being, offering actionable insights for the planning and design of similar urban waterfronts.

## Introduction

As urbanization shifts from “incremental expansion” to “stock quality improvement,” urban renewal has evolved beyond spatial renovation to become a national strategic priority^1^. Its core mission is to enhance urban development quality and meet public aspirations for improved living standards. Waterfront areas, unique zones where urban nature and culture intersect, serve as both crucial ecological nodes^2^ and vibrant spaces that foster high-quality living and social harmony^3^. Understanding the relationship between urban landscapes and vitality remains a key concern in contemporary urban studies, with recent scholarship continuing to refine core concepts of vitality such as activity intensity and diversity^4–7^. Consequently, research on waterfront public spaces has gained significant academic attention, and understanding their profound impact on sustainable urban development requires in-depth exploration.

A foundational challenge in waterfront research, first articulated by Jane Jacobs (1961), is the dialectical nature of water bodies, which can create “boundary vacuums” that sever the urban fabric even as they offer unique amenities^8^. This necessitates a focus beyond the internal design of waterfronts to their integration with the wider city. While existing studies have extensively analyzed linear riverfronts, exploring their impact on economic revitalization and public access^2^, the unique enclosed topology of urban lakeside areas and its influence on community-oriented activities remain under-explored. Methodologically, while novel techniques like GPS tracking and VR are reshaping waterfront research^9,10^, a critical gap persists in synthesizing these data-intensive approaches with classic observational frameworks, such as Roger Barker’s Behavior Setting Theory, which offers a robust lens for understanding the nuanced interplay between physical environments and standing patterns of behavior^11^.

**Fig. 1:**
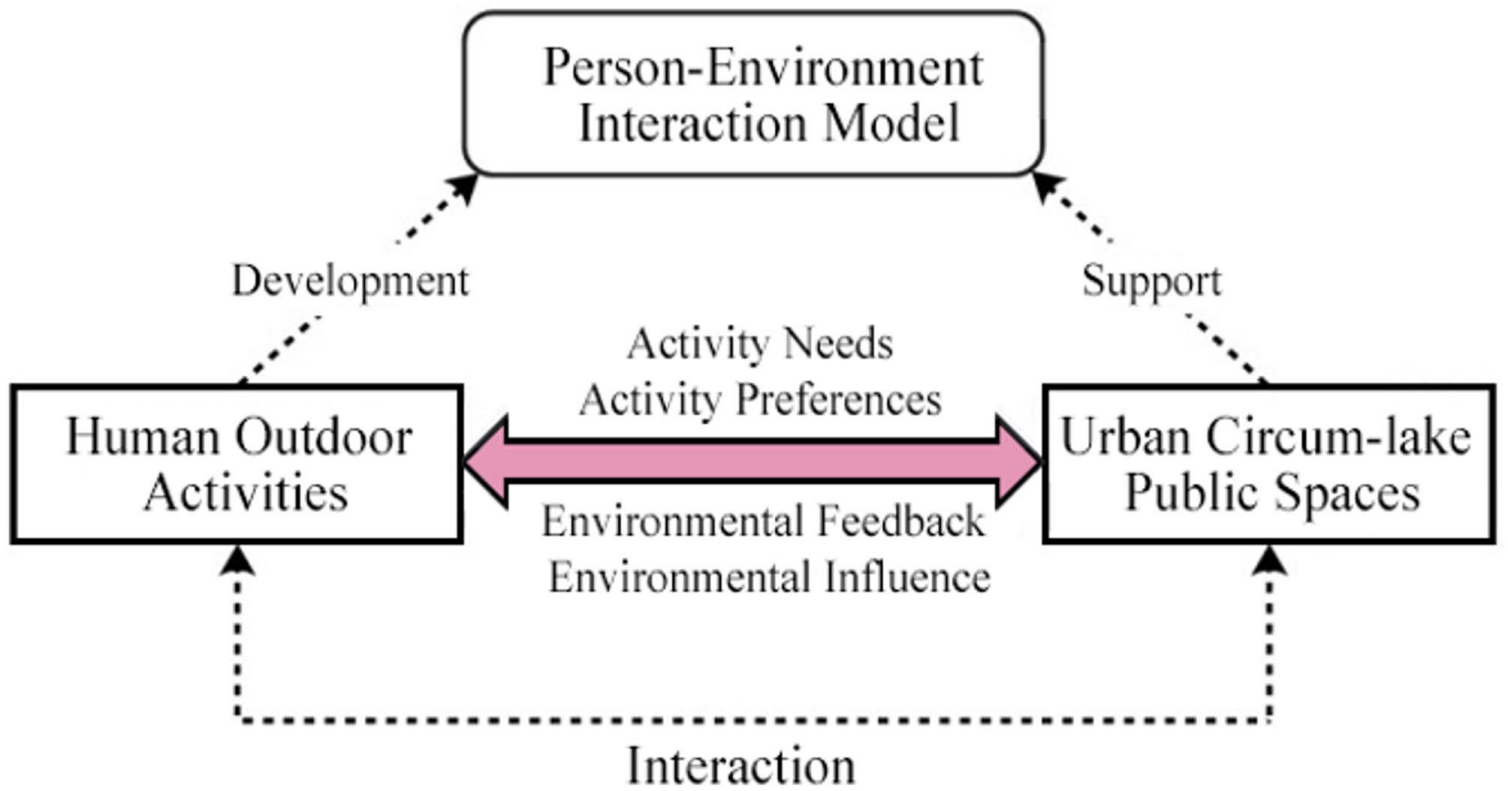
Conceptual model of the interaction between people and the public space surrounding the urban lake. Diagram illustrating the proposed “People-Public Space Surrounding the Lake” interaction model. It depicts the dynamic and reciprocal relationship where Human Outdoor Activities (influenced by activity needs and preferences) interact with Urban Public Spaces Surrounding the Lake (providing environmental affordances and feedback). The model highlights the ongoing processes of development, support, and mutual influence shaping both behavior and place.

To deconstruct this unique people-environment dynamic in lakeside settings, this study moves beyond the static concept of “place attachment,” which primarily focuses on an individual’s emotional bond with a place. We propose and empirically ground the concept of “place symbiosis”, defining it as a dynamic, co-evolutionary process where residents’activities continuously shape place meaning, and in turn, well-designed spaces afford and generate new interactions. While related to concepts like placemaking, “place symbiosis” distinctively emphasizes the spontaneous, adaptive, and reciprocal nature of everyday user-space interactions rather than predetermined planning interventions. Elucidating the pathways to achieving this “place symbiosis” is therefore the central aim of this research.Elucidațing the pathways to achieving this “place symbiosis,” as conceptualized in our interaction model (Fig. 1), is therefore the central aim of this research.

Therefore, using the area surrounding Nanchang’s Qingshan Lake as a case study, this research aims to systematically answer the following core research questions:

- RQ1: What are the unique spatio-temporal patterns of outdoor activities in urban lakeside spaces across different user groups?
- RQ2: How do different spatial typologies (area, line, and point) in lakeside public spaces afford and shape resident activities?
- RQ3: Through the lens of Behavior Setting Theory, what are the interaction mechanisms between residents’ adaptive behaviors and the lakeside micro-environment?
- RQ4: How can targeted micro-renewal strategies be developed to foster “place symbiosis” and enhance the vitality, inclusivity, and resilience of urban lakeside spaces?

## Methodology

### Study area

**Fig. 2:**
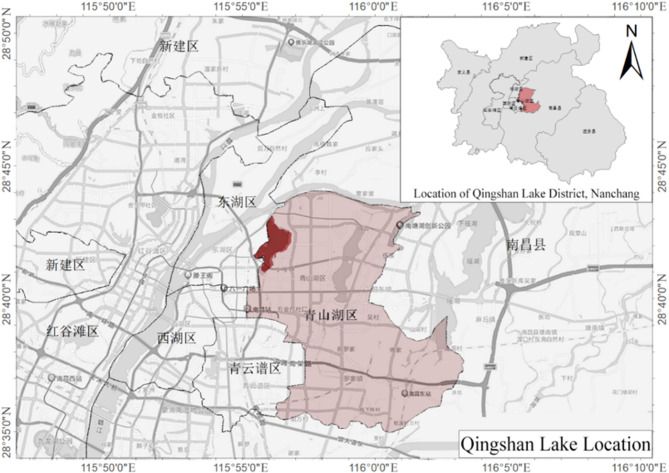
Location map of the Qingshan Lake study area. Map illustrating the location of Qingshan Lake within Nanchang city. It highlights the surrounding high-density residential areas, key districts, and the approximate 11 km shoreline encompassing the loop trail connecting various functional nodes. The primary study area focused on the public spaces immediately surrounding the lake is indicated.

Nanchang’s Qingshan Lake, situated in the city core, has a water area of 316 hectares and an approximate shoreline of 11 km. It is surrounded by high-density residential areas. A loop trail around the lake connects diverse functional nodes, including recreational squares and wetland parks, making this site an ideal case for studying the interaction between spaces surrounding the lake and resident behavior (Fig. 2). However, due to limitations from early development phases, the public spaces in this area exhibit shortcomings in layout, function, and quality. They currently struggle to meet residents’ growing demands for diverse, high-quality outdoor activities. Therefore, investigating the association between public space and resident behavior here is crucial for optimizing design, enhancing user experience, and providing a reference for similar areas surrounding urban lakes.

### Methodology

**Fig. 3:**
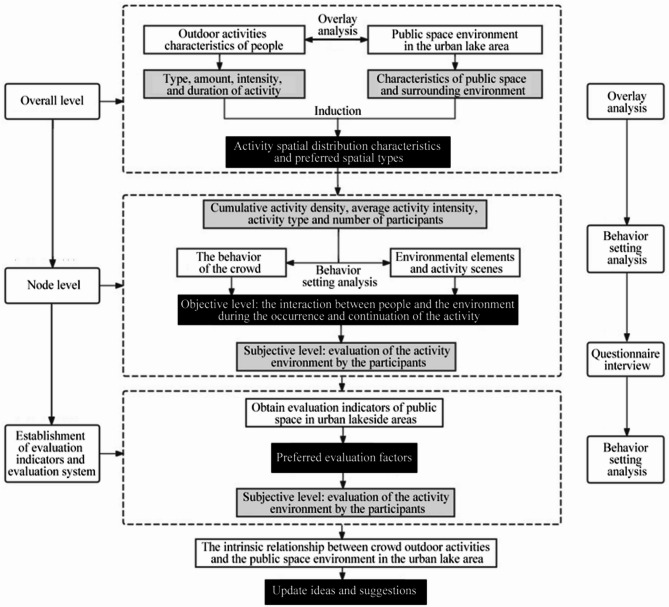
Multi-scale research framework. The framework illustrates an iterative and reciprocal analytical path. Initially, at the overall (macro) level, non-participant observation and GIS spatial analysis identify the general spatio-temporal distribution patterns of resident activities. These macro-level findings directly inform the selection of representative nodes for the micro-level analysis. Subsequently, at the node (micro) level, Behavior Setting Theory, behavior mapping, and subjective evaluations are integrated to investigate the mechanisms of people-environment interaction in depth. Finally, insights from the micro-level analysis are, in turn, used to explain and enrich the understanding of the macro-level spatial patterns, collectively leading to the formulation of targeted renewal strategies.

This study investigates the people-space relationship at both overall (macro) and node (micro) levels, utilizing a multi-scale analysis framework (Fig. 3). At the macro level, non-participant observation was conducted on clear weekdays and weekends. To mitigate potential biases from extreme midday conditions and correspondingly low activity levels, observation was concentrated during the more representative peak hours of the morning (07:00–12:00) and afternoon to evening (15:00–21:00), coupled with GIS spatial analysis to examine the spatio-temporal distribution patterns of activities and spatial preferences.

At the micro level, systematic behavior mapping^12^ was conducted at eight representative nodes. A trained team of four researchers ensured the rigor and consistency of this process, achieving high inter-rater reliability (Cohen’s Kappa > 0.85) through pre-study pilot tests. Over a two-week period on clear-weather days, the team conducted multiple 30-minute “snapshot” observation rounds at each node during peak activity hours. During each round, observers used GIS-based maps to precisely annotate users’ locations, activities, and demographic characteristics with a standardized set of symbols. Finally, residents’ subjective evaluations were integrated to provide a comprehensive understanding of the association between crowd activities and the public space environment surrounding the lake.

### Study population and sample size determination

Study participants were individuals active within the public spaces surrounding Qingshan Lake, categorized by age: minors (0–17 years), adults (18–59 years), and the elderly (≥ 60 years). Data collection involved 8 field surveys, yielding 384 valid activity records at the overall level (183 weekday, 201 weekend) and 313 valid activity records at the node level. Additionally, 200 questionnaires were distributed, resulting in 156 valid responses (a 78% response rate).

### Ethical considerations

This study was conducted in accordance with the principles of the Declaration of Helsinki. The research protocol, including procedures for data collection through non-participant observation and questionnaire surveys, received ethical approval from the Ethics Committee of the School of Architecture and Design, Nanchang University.

**Fig. 4:**
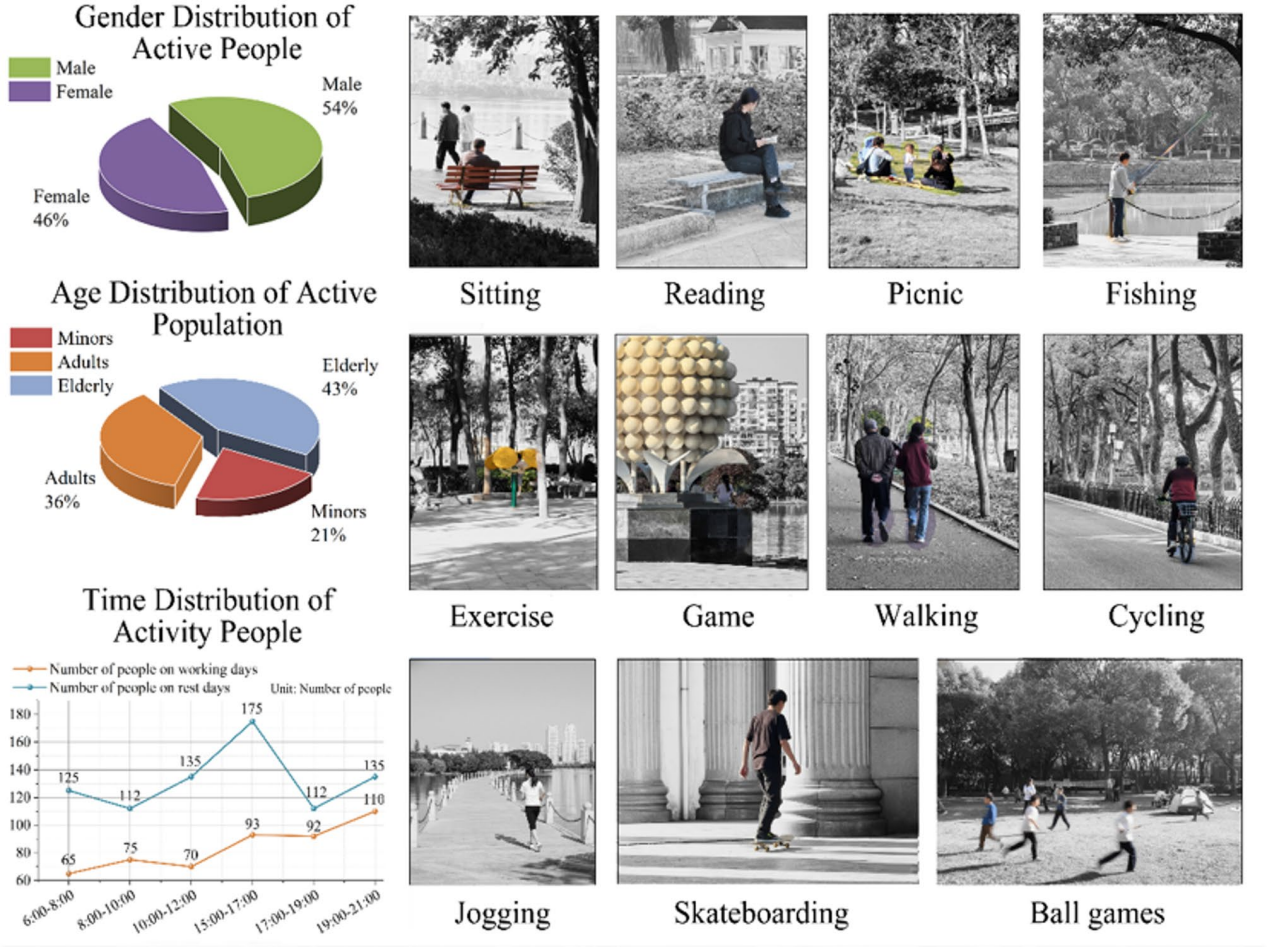
Characteristics of outdoor activity participants and activities around Qingshan Lake. Overview of the active population and their behaviors, showing: gender distribution of participants; age composition (Minors, Adults, Elderly), noting minors often accompanied by guardians; activity time distribution across different periods on weekdays and rest days; and photographic examples of the 11 main identified activity types (e.g., Sitting, Reading, Picnic, Fishing, Exercise, Game, Walking, Cycling, Jogging, Skateboarding, Ball games).

**Fig. 5:**
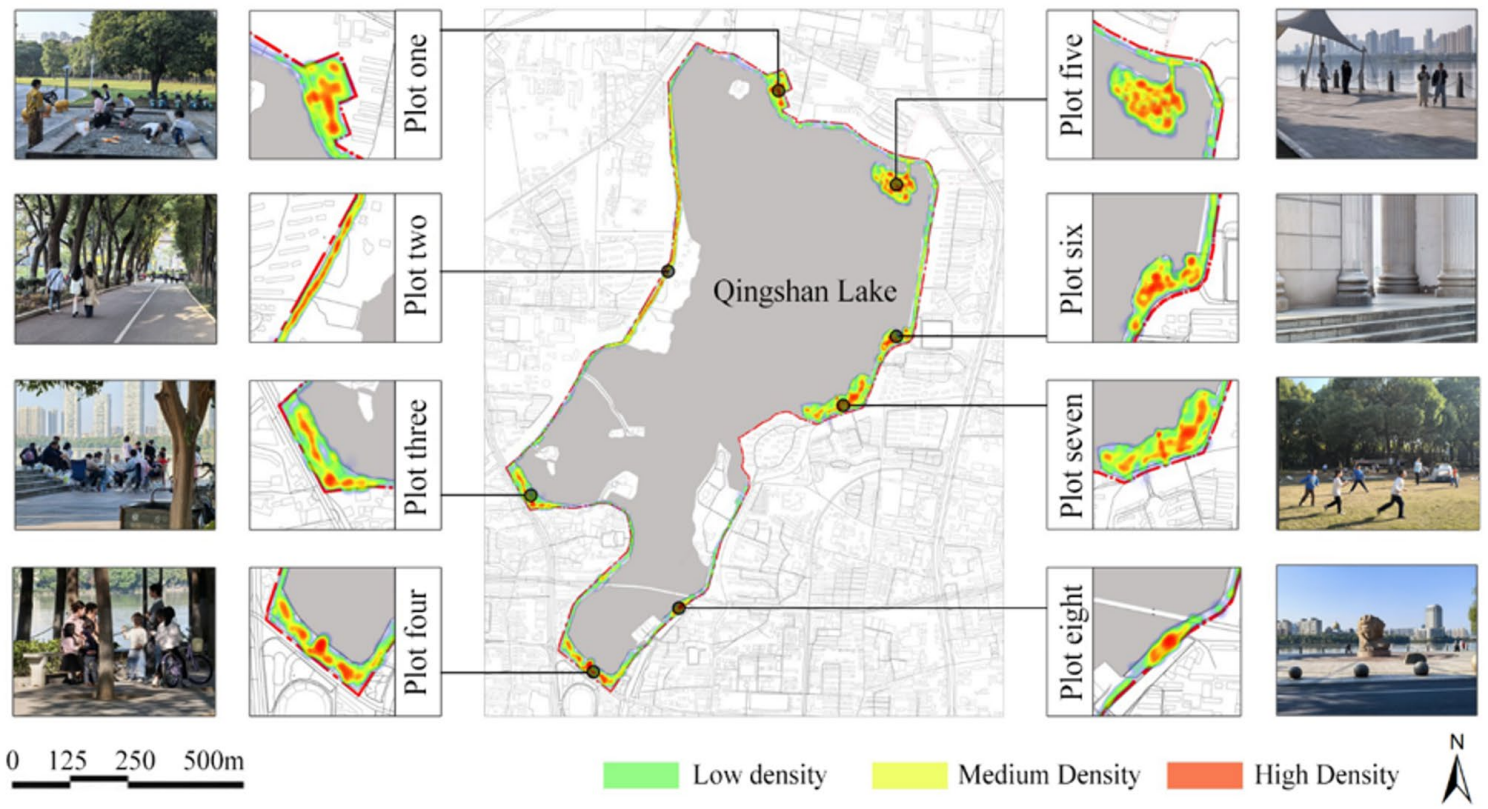
Spatial density distribution of outdoor activities around Qingshan Lake. Kernel density map generated using GIS spatial analysis, visualizing the concentration patterns of crowd outdoor activities. Warmer colors indicate higher activity density, revealing spatial variations such as core aggregation, scattered distribution, and linear extensions along pathways like the loop trail. Locations corresponding to the analyzed sample Plots (one through eight) are marked.

Informed consent was obtained from all individual participants included in the study. All adult participants provided informed consent before taking part in the survey. For participants under the age of 18, informed consent was obtained from their parents or legal guardians. Furthermore, specific written informed consent for the publication of images in an online open-access publication was obtained from all individuals whose faces are recognizably depicted in Figs. 4 and 5 (or from their legal guardians for minors). All participants were informed of the study’s objectives, the voluntary nature of their participation, and their right to withdraw at any time without consequence. Efforts were made to ensure the privacy and confidentiality of all participants.

## Spatio-temporal patterns of activity and public space use

### Overall level

#### Overview of crowd outdoor activities

The active population in the public spaces around Qingshan Lake primarily comprises adults (56%) and the elderly (25%); minors (19%) are often accompanied by guardians (Fig. 4). Activity peaks occur between 6:00–10:00 and 14:00–18:00, with maximum density observed between 10:00–12:00, followed by another rise after 15:00 continuing until 21:00. Among the 11 main activity types identified, sedentary activities (sitting, strolling, picnicking) and running constitute a significant proportion (approx. 60%), followed by equipment-based fitness, cycling, and ball games. Significant age-related differences exist: elderly individuals typically prefer low-intensity activities (e.g., sitting, strolling, equipment-based fitness), adults engage more in moderate-to-high intensity activities (e.g., running, cycling), and minors favor recreational pursuits (e.g., interacting with small features, playing ball games).

#### Spatial distribution characteristics of crowd activities and preferred space types

Using a GIS platform, kernel density analysis generated a spatial density distribution map of crowd outdoor activities (Fig. 5). Overlay analysis with the area’s environmental characteristics revealed that variations in site spatial morphology significantly influence resident activity distribution, leading to distinct spatial zones with unique features. Within these zones, activity distribution patterns vary (e.g., core aggregation, scattered distribution, linear extension along pathways), forming unique small-scale spatial structures. Despite this zoning, the lake loop trail is crucial for connecting activity spaces across zones and enhancing the area’s overall coherence. Resident activity spaces were categorized into three types: point, line, and area. Area-type spaces (e.g., squares, parks) are typically open areas characterized by gathering potential and openness. Line-type spaces usually follow paths like the lake loop trail, forming informal activity zones characterized by fluidity and temporality. Point-type spaces primarily consist of stationary, dispersed elements such as leisure seating, pavilions, and architectural features.

#### Transition from macro patterns to Micro-Level nodes

The preceding macro-level analysis reveals a significant spatial heterogeneity in activities within the public spaces surrounding Qingshan Lake, characterized by zones of core aggregation, linear extension, and scattered distribution. However, the underlying causes of these macro patterns—namely, why residents prefer certain spaces and how they specifically interact with the environmental features therein—require in-depth micro-level investigation. Therefore, to uncover the mechanisms driving these macro-level phenomena, we selected eight typical nodes based on the varying activity densities and spatial typologies (area-type, line-type, and point-type) identified in the macro-analysis. This targeted selection transitions the study to the subsequent node-level analysis.

### Node level

#### Selection and typology of representative nodes

**Fig. 6:**
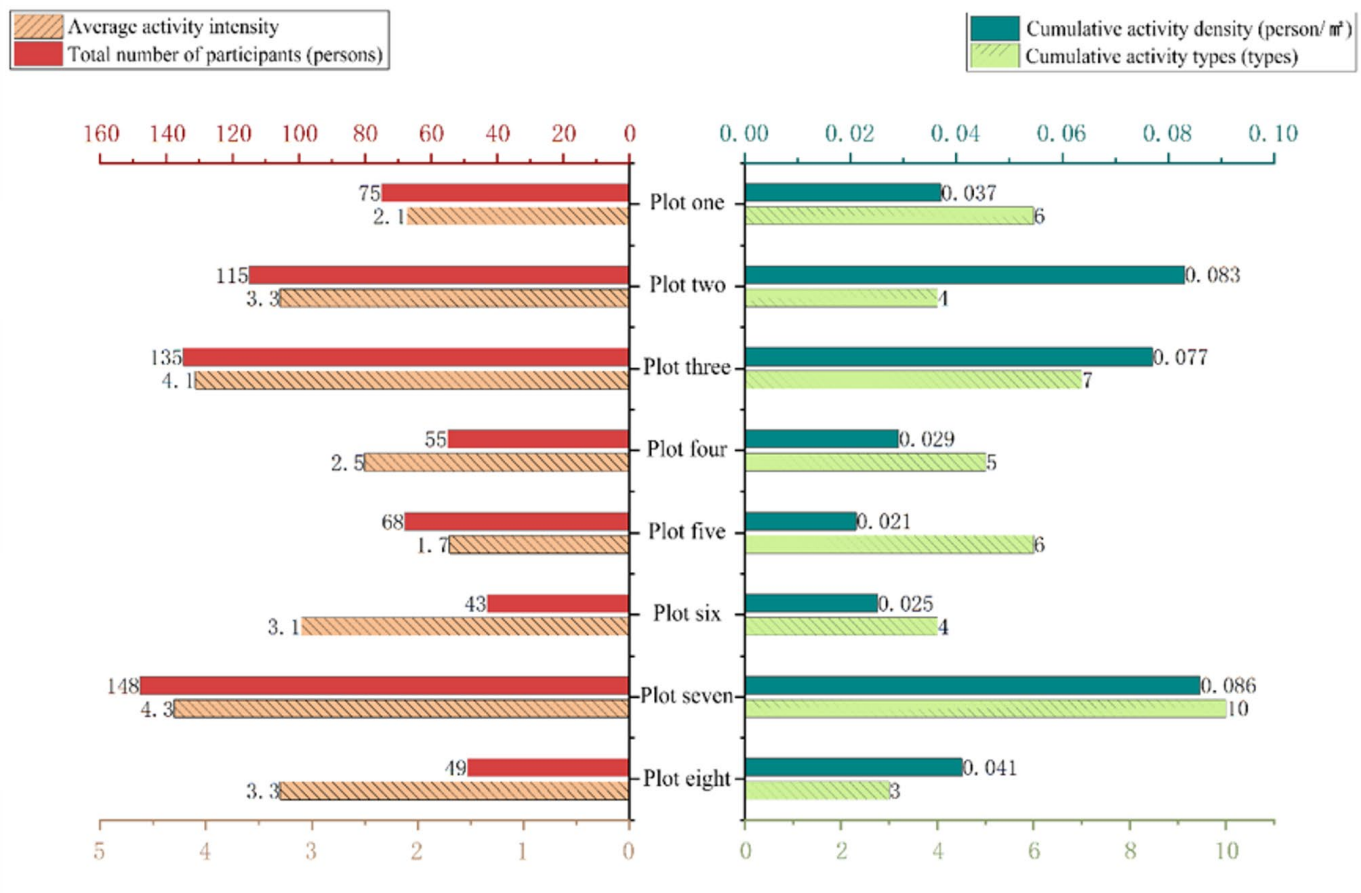
Comparative analysis of activity characteristics across representative node spaces. Bar charts comparing the eight selected representative node spaces (Plots 1–8) based on four activity metrics derived from field observations: average activity intensity (subjectively rated on a 1–5 scale), total number of participants recorded, cumulative activity density (participants per square meter), and cumulative number of distinct activity types observed.

To ensure representativeness, our micro-level analysis focused on 8 nodes selected through a systematic, multi-stage process. First, a GIS-based inventory identified a sampling frame of 25 candidate nodes based on quantifiable spatial criteria (e.g., area > 500 m², ≥ 3 path intersections). These candidates were then ranked using a composite Vitality Index, which integrated four normalized indicators from our macro-level observations: density, intensity, diversity, and participant numbers. Finally, the 8 representative nodes were selected via a stratified purposive sampling strategy. This involved first stratifying the ranked nodes by spatial typology (Area-type, Line-type, and Point-type) and then purposefully selecting the highest-vitality exemplars from each stratum to ensure typological and spatial diversity. A comparative analysis of the key activity characteristics for these eight selected nodes is presented in (Fig. 6), illustrating the variations in vitality that informed their selection.

#### Interaction between activities and the node environment

Empirical research reveals a dynamic interaction between residents and the environmental elements of the public space surrounding the lake (Table 1). Residents not only utilize the space’s intended functions but also adaptively use and effectively “redefine” it according to their needs. For instance, the lakefront square (a specific type of space at the edge), a typical composite activity site, shows clear temporal functional shifts: daytime use primarily supports fitness and parent-child activities (e.g., running, exercise, kite flying), while nighttime transforms the area into a primary venue for middle-aged and elderly group dancing, offering diverse cultural and entertainment opportunities. Similarly, residents utilize flexible open spaces (e.g., lawns, paved areas) to temporarily set up stages, tents, and stalls for community events like festivals or markets, reflecting the space’s adaptability and significantly enhancing community cohesion. Regarding resting facilities, beyond traditional use of seats, pavilions, and lawns, residents creatively adapt these for activities like outdoor picnics, reading, and socializing (e.g., using chess tables integrated into social nodes). This adaptive use enriches the functional capacity of the public space and strengthens residents’ sense of place and belonging, integrating the environment surrounding the lake into their daily lives. These instances of user-led spatial redefinition are tangible manifestations of the dynamic people-space relationship, providing direct evidence for the process of place symbiosis.

**Table 1:** Relationship between public space Elements, behavioral Support, and functional effects in the public space surrounding Qingshan Lake, Nanchang.

Core Design Orientation	Key Design Elements and Facilitated Activities	Main Functions and Effects
Enhancing Spatial Vitality & Diversity	1. Composite Activity Sites (Squares/Open Areas): Parent-child activities, group activities (square dancing, etc.). 2. Themed Functional Nodes (Sports/Play Areas): Equipment-based fitness, ball games, children’s play, group gatherings.	1. Enhances urban public space vitality; 2. Promotes multicultural integration; 3. Meets diverse population activity needs; 4. Provides cultural and entertainment options.
Enhancing Site Adaptability & Mixed Use	1. Flexible Open Spaces: Accommodate temporary/seasonal activities, support spontaneous large gatherings (festivals/markets), campsite setups (tents), and informal sports. 2. Temporary Activity Support: Enables setup of stages, market stalls, etc., on weekends/holidays.	1. Enhances social cohesion; 2. Promotes community interaction; 3. Strengthens community identity; 4. Meets event hosting needs; 5. Reflects spatial usage flexibility.
Optimizing Rest Experience & Social Environment	1. Diverse Resting Nodes (Seats/Pavilions/Steps/Lawns): Provide varied opportunities for static lingering, viewing, reading, informal communication. 2. Amenity-Rich Environmental Elements (Greenery/Waterscapes/Shade): Create comfortable, relaxing microclimates, attract people to stay, enhance sensory experience. 3. Social Node Design (Chess tables/Gathering points/Small plazas): Encourage close interaction, neighborly exchange, and small group activities (chess/picnics).	1. Meets diverse resting needs; 2. Improves place environmental comfort; 3. Fosters sense of belonging & community identity; 4. Promotes informal social interaction; 5. Enriches public space functions; 6. Builds positive social interaction settings.

This table summarizes the observed relationships between core design orientations (e.g., Enhancing Vitality), key environmental elements or design features present in the Qingshan Lake public spaces, the specific outdoor activities these elements facilitate, and the resulting main functions or effects on the user experience and space characteristics. Examples are drawn from field observations within the study area.

To systematically understand these interactions, we employed Behavior Setting Theory. This revealed that different environmental settings—such as rest areas, fitness zones, and pathways—foster distinct “behavior settings” where activity types vary according to spatial attributes (Table 2). Consequently, we classified the observed activities into three levels of intensity:

**Table 2:** Analysis of outdoor activity intensity type characteristics in urban public space surrounding Lakes.

Category	Activity Population	Activity Classification	Activity Space	Spatial Role	Environmental Preference	Facility Needs	Social Attributes
Low-Intensity Activities	Middle-aged/Elderly, Couples, Picnickers	Sitting, chess, picnics, fishing	Sites rich in vegetation, open views (often with seats, lawns, pavilions)	Relaxation, leisure, social venue	Quiet, rich vegetation, open views	Seats, pavilions, lawns, etc.	Individual/ Small group oriented
Medium-Intensity Activities	Middle-aged/Young Adults, Parent-child families, Group residents	Square dancing, skateboarding, strolling, Tai Chi	Larger sites (or surroundings often equipped with fitness gear)	Multi-functional, inclusive, meets diverse needs	Open, flat, equipment-accessible	Fitness equipment, flat ground	Group-oriented, high interactivity
High-Intensity Activities	Sports Enthusiasts	Ball games, jogging, cycling	Loop trails, cycle paths, professional sports courts	Professional function, competitive, meets high standards	Professional sites, open, safe	Professional courts/equipment, trails, cycle paths	Individual/ Group, competitive

This table categorizes observed outdoor activities into three intensity levels (Low, Medium, High) based on behavioral characteristics. For each level, it details typical participant demographics (Activity Population), common activities (Activity Classification), typical spatial settings (Activity Space), the role these spaces play (Spatial Role), preferred environmental qualities (Environmental Preference), necessary physical infrastructure (Facility Needs), and common social interaction patterns (Social Attributes).

- **Low-Intensity Activities**: Characterized by people in resting or slow-moving states, with limited activity range. Participants are mainly those resting, middle-aged/elderly individuals, couples, and picnickers. These spaces are often located in areas rich in vegetation with open views, equipped with resting facilities (e.g., seats, lawns, pavilions), emphasizing comfort and leisure.
- **Medium-Intensity Activities**: Characterized by people in relatively active states, with a moderate activity range. Participants include those exercising, parent-child families, and group activity participants. Spaces are often open and flat, accommodating larger groups for activities like equipment-based fitness, square dancing, and ball games. Fitness equipment is often available nearby, emphasizing multi-functionality and inclusivity.
- **High-Intensity Activities**: Characterized by people engaged in high-intensity exercise, covering a larger range. Participants are mainly sports enthusiasts, and spaces are often loop trails or cycle paths. These sites not only offer ample space but also feature suitable ground materials (e.g., rubber tracks) for sports, providing a safe and efficient exercise environment, emphasizing professionalism and functionality.

#### Subjective evaluation of node activity space environment

**Fig. 7:**
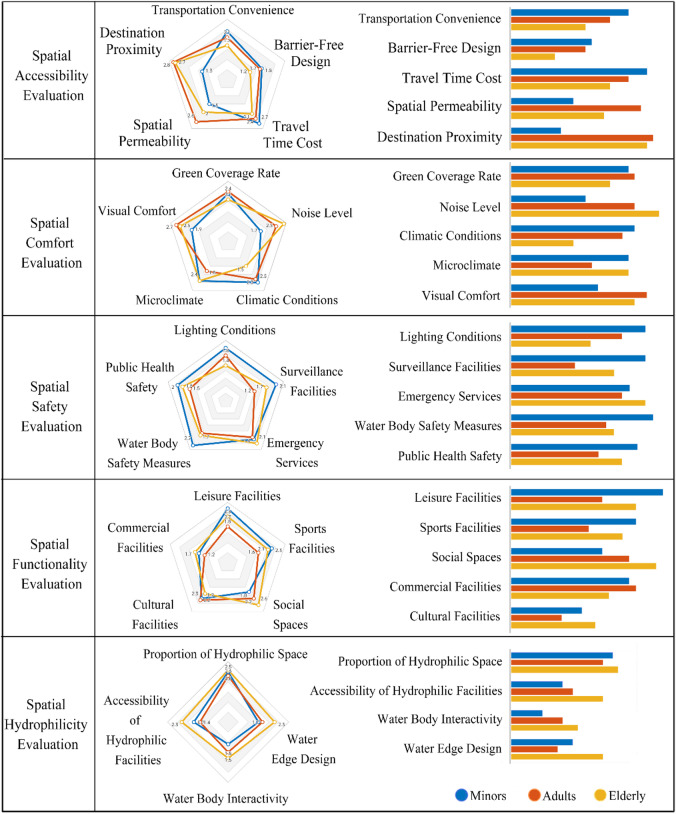
User satisfaction evaluation of node activity space environment by age group. Radar charts (left) and corresponding detailed bar charts (right) illustrating the subjective satisfaction scores from different age groups (Minors, Adults, Elderly) across five evaluation dimensions (Spatial Accessibility, Comfort, Functionality, Safety, Hydrophilicity) and their 24 constituent secondary indicators. Scores are based on a three-point Likert scale (1=Dissatisfied, 2=Neutral, 3=Very Satisfied) survey.

Based on on-site questionnaire surveys and referencing previous studies^13^, we developed an evaluation system comprising five dimensions: Spatial Accessibility, Comfort, Functionality, Safety, and Hydrophilicity, encompassing 24 indicators. A three-point Likert scale (1 = Dissatisfied, 2 = Neutral, 3 = Very Satisfied) was used for scoring. Average scores for each indicator were calculated (Fig. 7) to provide a basis for subsequent analysis. Radar chart analysis revealed significant differences in satisfaction levels across age groups. Minors reported higher satisfaction with spatial accessibility, comfort, and functionality, particularly rating the natural environment, terrain variations, safety, and challenge aspects positively. Conversely, adults and the elderly expressed lower satisfaction regarding safety and comfort, primarily due to issues such as vehicle management at entrances/exits, lack of spatial surveillance in concealed areas, insufficient nighttime lighting, and seasonal climate discomfort. A common desire across all age groups was for additional recreational and entertainment facilities. These findings suggest that the public spaces around Qingshan Lake require improvements to better meet the specific needs of adult and elderly users.

## Evaluation study

### Evaluation system construction and indicator selection

**Fig. 8:**
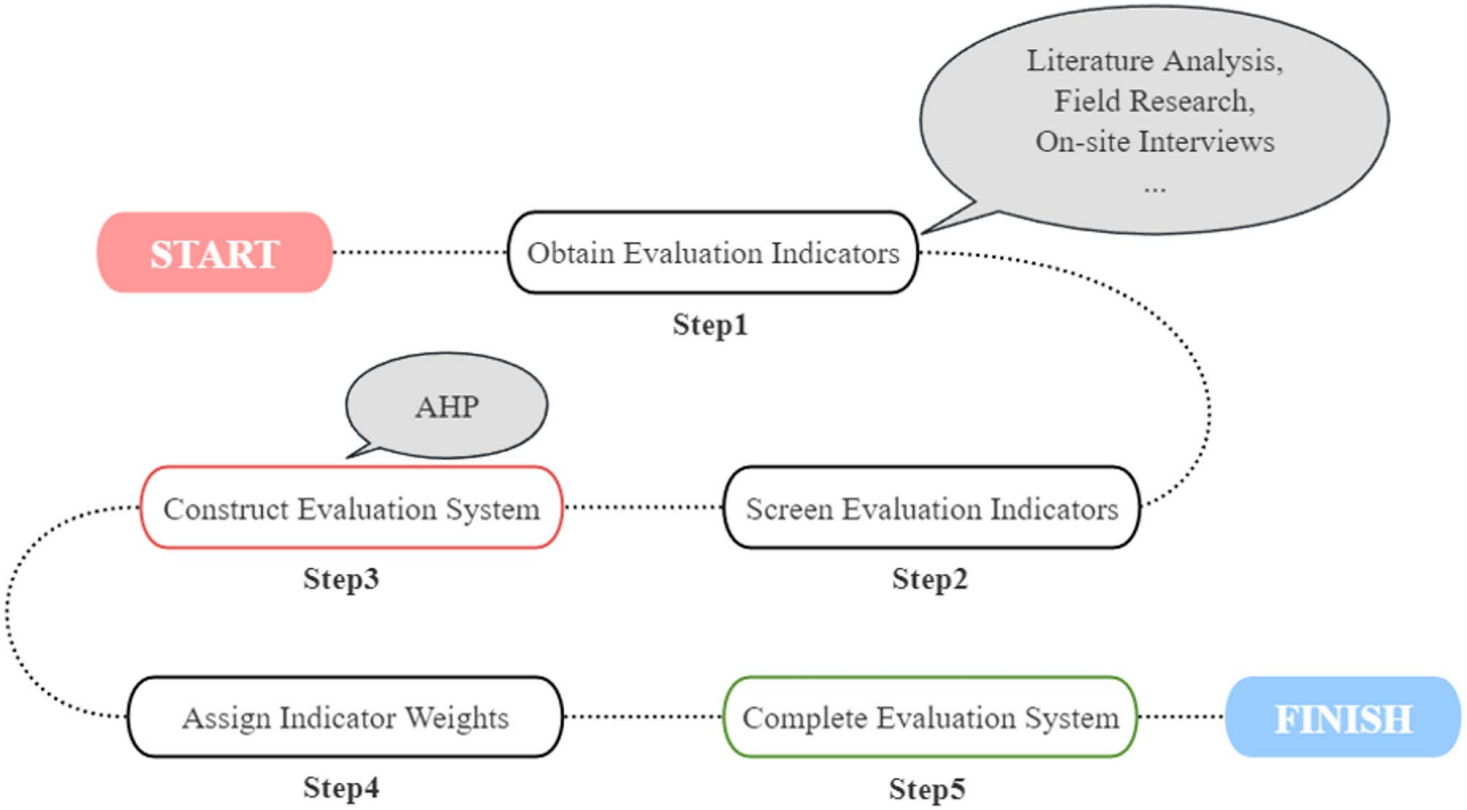
Structure and construction process of the AHP evaluation system. Flowchart detailing the methodology for constructing the four-level Analytic Hierarchy Process (AHP) evaluation system used in this study. Steps include: Step 1 - Obtain Evaluation Indicators (from literature, surveys, etc.); Step 2 - Screen Evaluation Indicators; Step 3 - Construct Evaluation System hierarchy (Goal, Criteria/Primary Indicators, Indicators/Secondary Indicators); Step 4 - Assign Indicator Weights (using AHP); Step 5 - Complete and apply the Evaluation System.

To quantitatively evaluate the association between the public space around Qingshan Lake and resident activity behavior, we developed a comprehensive evaluation system. Integrating theoretical research, field surveys, literature analysis, and expert consultation, key indicators reflecting the link between resident activity behavior and spatial characteristics in the study area were selected. The Analytic Hierarchy Process (AHP)^14^ was used to quantify evaluation elements and construct a four-level system comprising: Goal, Criteria (Primary Indicators), and Indicators (Secondary Indicators). Combined with questionnaire data on indicator importance and weight assignment, a complete evaluation system was established (Fig. 8). This system is scientifically sound, feasible for assessing the environmental impact on resident activities around Qingshan Lake, and provides a foundation for related research.

Drawing on theory and empirical findings, we selected indicators influencing the association between resident activity behavior and spatial characteristics around Qingshan Lake. A four-level evaluation system was constructed (Fig. 8), comprising the Goal layer, Criteria layer (Primary Indicators), and Indicator layer (Secondary Indicators), designed to comprehensively capture key spatial dimensions affecting resident use. The specific indicators are:

- **Goal Layer**: Evaluation of the Association between Resident Outdoor Activity Behavior and the Space Surrounding the Lake.
- **Primary Indicators (5)**: Spatial Accessibility, Spatial Comfort, Spatial Functionality, Spatial Safety, Spatial Hydrophilicity.
- **Secondary Indicators (24)**: Detailed indicators elaborated under each primary indicator (see below).

**(a) Spatial Accessibility Evaluation**:

(1) Transportation Convenience: Assessing public transport coverage, road network density, parking facilities, etc.

(2) Barrier-Free Design: Evaluating the adequacy of accessible pathways, ramps, tactile paving, accessible restrooms, and signage systems within the space.

(3) Travel Time Cost: Average travel time from residential areas to the public space.

(4) Spatial Permeability: Evaluating the number, location, and opening hours of public space entrances, and whether they are open to all user groups.

(5) Destination Proximity: Straight-line and actual walking distance for residents to reach the public space, and whether crossing unsafe areas is required.

**(b) Spatial Comfort Evaluation**:

(1) Green Coverage Rate: Degree of vegetation cover within the public space, affecting air quality and visual comfort.

(2) Noise Level: Assessing the level of noise pollution within the public space and its impact on resident activities.

(3) Climatic Conditions: Impact of temperature, humidity, wind speed, etc., on residents’ outdoor activities.

(4) Microclimate: Evaluating microclimatic conditions in different areas, such as the distribution of shaded and sunny areas.

(5) Visual Comfort: Assessing the aesthetic appeal of the public space, including landscape design, art installations, and color schemes.

**(c) Spatial Functionality Evaluation**:

(1) Leisure Facilities: Provision of seating, pavilions, children’s play facilities, etc., within the public space.

(2) Sports Facilities: Such as trails, cycle paths, sports fields, etc.

(3) Social Spaces: Squares, gathering points, etc., provided for resident interaction.

(4) Commercial Facilities: Assessing the distribution and diversity of commercial facilities within the public space, such as cafes, restaurants, and shops.

(5) Cultural Facilities: Evaluating the availability of cultural facilities within the public space, such as libraries, exhibition halls, and performance spaces.

**(d) Spatial Safety Evaluation**:

(1) Lighting Conditions: Adequacy of nighttime lighting, affecting residents’ sense of security.

(2) Surveillance Facilities: Coverage range and density of surveillance cameras.

(3) Emergency Services: Accessibility of emergency medical services, safety exits, etc.

(4) Water Body Safety Measures: Considering safety measures around water bodies, such as railings, life-saving equipment, warning signs, etc., to ensure the safety of residents’ hydrophilic activities along the lake edge.

(5) Public Health Safety: Evaluating public health facilities within the public space, such as handwashing facilities, waste recycling stations, and cleaning services.

**(e) Spatial Hydrophilicity Evaluation**:

(1) Proportion of Hydrophilic Space: Assessing the proportion of hydrophilic areas (e.g., lake edge trails, waterfront platforms, piers) within the total public space of the area surrounding Qingshan Lake.

(2) Accessibility of Hydrophilic Facilities: Considering the distribution density and accessibility of hydrophilic facilities (e.g., seating, pavilions, viewing platforms), and whether they facilitate direct contact with the water body.

(3) Water Body Interactivity: Evaluating the interactivity between the water body and resident activities in the public space, such as whether facilities for boating, fishing, etc., are provided.

(4) Water Edge Design: Evaluating whether the design of the water edge encourages people to approach the water, e.g., presence of steps, ramps, floating docks, etc., which can increase interaction with water.

### Determining evaluation indicator weights

**Fig. 9:**
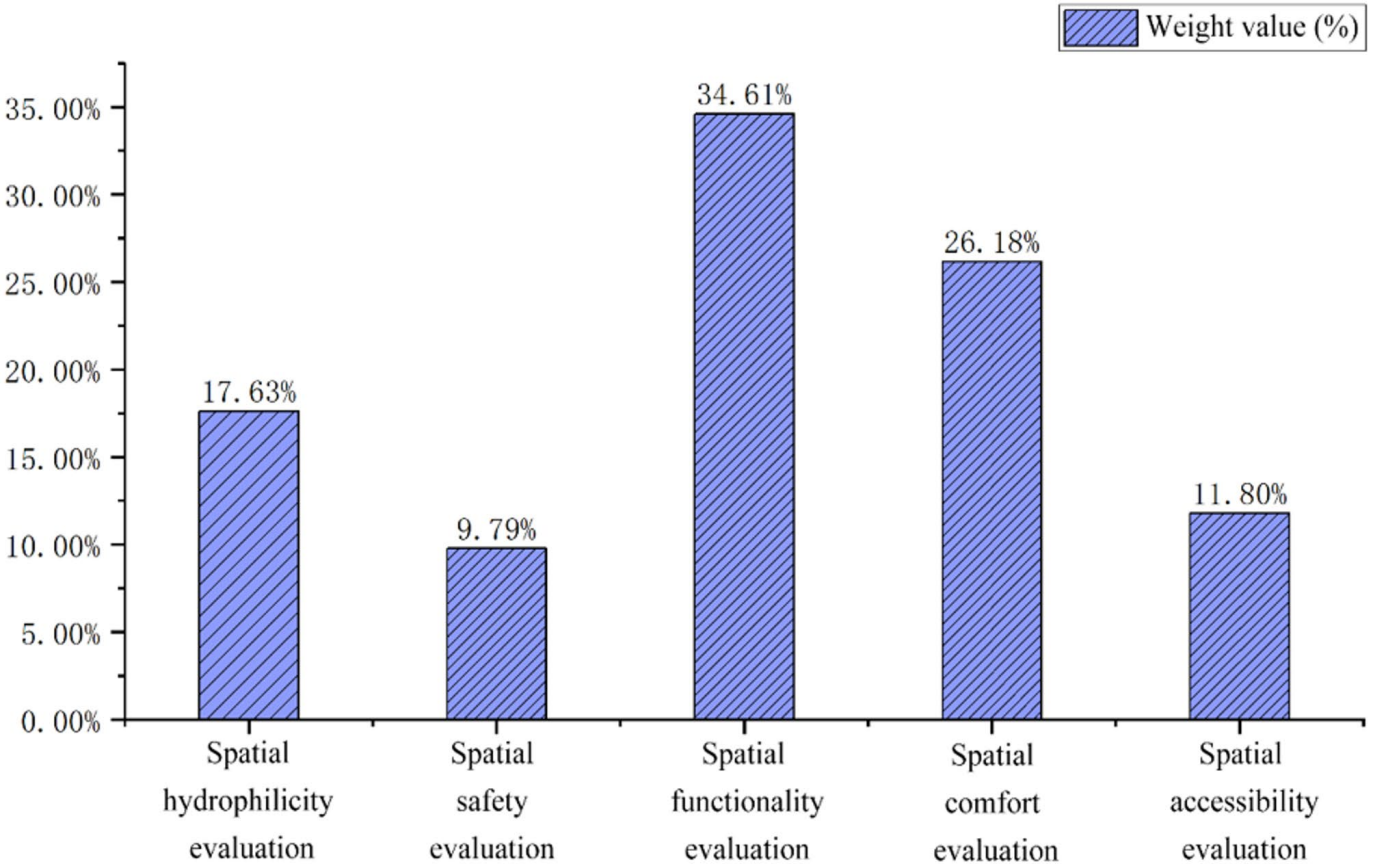
Weight distribution of primary evaluation indicators determined by AHP. Bar chart displaying the calculated relative weights (as percentages) for the five primary evaluation indicators (Spatial Accessibility, Comfort, Functionality, Safety, and Hydrophilicity). These weights were derived from the Analytic Hierarchy Process (AHP) analysis based on questionnaire and interview data regarding perceived importance.

To construct the AHP hierarchy model, a 5th-order judgment matrix was built for the five primary indicators: Spatial Accessibility, Comfort, Functionality, Safety, and Hydrophilicity. Based on questionnaire and interview data, the sum-product method was used to calculate the scores for each evaluation factor, analyzed using AHP software. The resulting eigenvector was (0.590, 1.309, 1.730, 0.490, 0.882), corresponding to weights of 11.796%, 26.177%, 34.606%, 9.791%, and 17.630% respectively (Fig. 9). The maximum eigenvalue (λmax) was 5.405.

For this 5th-order judgment matrix, the corresponding Random Consistency Index (RI) from standard tables (Table 3) is 1.120. This RI value was used for the consistency check. The Consistency Index (CI) was calculated as CI = (λmax - n)/(*n* − 1) = (5.405–5)/(5 − 1) = 0.101 (for *n* = 5). The Consistency Ratio (CR) was then determined as CR = CI/RI = 0.101/1.120 = 0.090. Since CR (0.090) is less than the threshold of 0.1, the judgment matrix satisfies the consistency check, indicating that the calculated weights are consistent (Table 4).

**Table 3:** Random consistency RI table.

*n*	3	4	5	6	7	8	9	10	11	12	13	14	15	16
RI	0.52	0.89	1.12	1.26	1.36	1.41	1.46	1.49	1.52	1.54	1.56	1.58	1.59	1.5943
*n*	17	18	19	20	21	22	23	24	25	26	27	28	29	30
RI	1.6064	1.6133	1.6207	1.6292	1.6358	1.6403	1.6462	1.6497	1.6556	1.6587	1.6631	1.6670	1.6693	1.6724

Standard Random Consistency Index (RI) values corresponding to the order (n) of the judgment matrix in the Analytic Hierarchy Process (AHP). These values are used to calculate the Consistency Ratio (CR) to check the consistency of pairwise comparisons. Values are derived from Saaty’s standard RI tables.

**Table 4:** Consistency check results summary.

Maximum Eigenvalue (λmax)	CI	RI	CR	Consistency Check Result
5.405	0.101	1.120	0.090	Passed

Results of the consistency check for the 5th-order AHP judgment matrix comparing the primary evaluation indicators. *λmax *  Maximum Eigenvalue, *CI* Consistency Index, calculated as (λmax - n)/(*n* − 1), *RI* Random Consistency Index (for *n* = 5 from Table 3), *CR* Consistency Ratio, calculated as CI/RI. A CR value less than 0.10 indicates that the pairwise judgments are acceptably consistent.

### Evaluation results

The results (Table 5) indicate that Spatial Functionality (weight: 34.61%) is the dimension of greatest concern to residents, weighted significantly higher than others. This suggests that residents’ strong demand for diverse activities and facilities around the lake is the primary factor influencing their evaluation. Comfort (26.18%) and Hydrophilicity (17.63%) follow, reflecting the importance placed on environmental quality and the near-water experience. Accessibility (11.80%) holds relatively lower importance. Notably, Safety (9.79%) has the lowest relative weight. This may indicate that respondents perceive the overall safety conditions around Qingshan Lake as generally acceptable, or that once basic security needs are met, focus shifts towards functional satisfaction and environmental experience. This does not diminish the importance of safety; rather, it highlights the need for precise solutions to specific issues (e.g., lighting, waterside protection). Analysis of secondary indicators reveals key drivers within each dimension, such as leisure and sports facilities (under Spatial Functionality) and green coverage rate (under Spatial Comfort), which are critical focal points for refined public space design.

**Table 5:** Summary of evaluation results.

Category	Weight (%)	Eigenvector	Node item	Weight (%)	Eigenvector	Consistency check
Spatial Accessibility Eval	11.80%	0.59	Transportation Convenience	19.55%	0.115	Passed
			Barrier-Free Design	9.23%	0.054	Passed
			Travel Time Cost	39.77%	0.235	Passed
			Spatial Permeability	15.45%	0.091	Passed
			Destination Proximity	15.99%	0.094	Passed
Spatial Comfort Eval	26.18%	1.309	Green Coverage Rate	39.22%	1.026	Passed
			Noise Level	7.83%	0.205	Passed
			Climatic Conditions	34.48%	0.902	Passed
			Microclimate	7.29%	0.191	Passed
			Visual Comfort	11.18%	0.293	Passed
Spatial Functionality Eval.	34.61%	1.73	Leisure Facilities	34.79%	1.205	Passed
			Sports Facilities	35.21%	1.219	Passed
			Social Spaces	17.48%	0.605	Passed
			Commercial Facilities	6.93%	0.240	Passed
			Cultural Facilities	5.59%	0.194	Passed
Spatial Safety Eval.	9.79%	0.49	Lighting Conditions	39.48%	0.193	Passed
			Surveillance Facilities	9.77%	0.048	Passed
			Emergency Services	24.79%	0.121	Passed
			Water Body Safety Measures	19.93%	0.098	Passed
			Public Health Safety	6.03%	0.030	Passed
Spatial Hydrophilicity Eval.	17.63%	0.882	Proportion of Hydrophilic Space	27.53%	0.243	Passed
			Accessibility of Hydro. Facil	44.48%	0.392	Passed
			Water Body Interactivity	11.79%	0.104	Passed
			Water Edge Design	16.20%	0.143	Passed

Results of the AHP evaluation showing calculated weights (%) and eigenvectors for primary indicators (Category) and secondary indicators (Node Item) across the five dimensions. The ‘Weight (%)’ column indicates the relative importance of each indicator. ‘Consistency Check’ confirms that the pairwise comparisons for the secondary indicators within each primary category passed the consistency test (CR < 0.1). *Eval* Evaluation, *Hydro. Facil* Hydrophilic Facilities.

Notably, the Safety dimension (9.79%) has the lowest relative weight among the five dimensions. This finding should not be interpreted as safety being unimportant; on the contrary, it is the fundamental prerequisite for all public space use. This seemingly counter-intuitive result reveal a deeper logic of user perception, reflecting Maslow’s hierarchy of needs^15^ in built environment evaluation: once a baseline level of safety is perceived as adequate (fulfilling “Physiological and Safety” needs), users’ focus naturally shifts towards higher-order dimensions like Functionality and Comfort that directly enhance their experience (fulfilling “Social and Esteem” needs). However, this macro-level weighting does not mask specific micro-level safety concerns. As detailed in the subjective evaluations, strong user concern regarding specific issues like “Lighting Conditions” persists. This highlights a critical gap between baseline safety and a high-quality sense of security, which is precisely the leverage point our renewal strategies must address.

#### Spatial accessibility evaluation

Spatial accessibility fundamentally affects residents’ convenience in using the public space surrounding the lake and forms a basic layer of the evaluation system. Within this dimension, travel time cost emerged as the most crucial factor, reflecting residents’ strong emphasis on travel efficiency. Compared to travel time, transportation convenience and the provision of barrier-free facilities were weighted lower. These results suggest that enhancing public space accessibility should prioritize optimizing the transportation network to reduce travel times, while concurrently strengthening barrier-free design and construction to improve spatial inclusivity for diverse user groups.

#### Spatial comfort evaluation

Spatial comfort, ranking second in overall weight, is a core dimension influencing residents’ experience and satisfaction within the environment surrounding the lake. Green coverage rate is the dominant factor within this dimension, reflecting high expectations for the extent and quality of vegetation in public spaces. Climatic conditions also significantly affect comfort. While green coverage and climate are primary drivers, the influence of noise levels, though weighted lower, requires attention, particularly in areas adjacent to high-density residential zones.

#### Spatial functionality evaluation

Ranking highest in weight, Spatial Functionality is the primary dimension determining resident satisfaction with the public space surrounding the lake, directly reflecting the core demand for diverse activities and facilities. Within this dimension, leisure and sports facilities are the elements of greatest importance to residents, indicating strong expectations for public spaces to meet recreational and fitness needs. While weighted lower, social spaces remain an indispensable component of overall public space functionality.

#### Spatial safety evaluation

Spatial Safety ranked relatively low in the overall weighting. This likely reflects that, assuming basic safety is met, residents prioritize other dimensions, rather than diminishing the fundamental importance of safety for public space use. Within this dimension, lighting conditions received the most weight, highlighting concerns about nighttime activity safety. Emergency service accessibility and waterside safety measures also significantly influenced safety perceptions. Conversely, the weight assigned to surveillance facilities was lower. These findings suggest that efforts to improve public space safety should prioritize adequate lighting, accessible emergency services, and enhanced safety measures along the lake edge.

#### Spatial hydrophilicity evaluation

Spatial Hydrophilicity, reflecting the unique character of the area surrounding the lake and ranking third in weight, highlights the importance residents place on the near-water experience. Within this dimension, the accessibility of hydrophilic facilities is the primary consideration, indicating high expectations for convenient access to water-adjacent spaces. The proportion of hydrophilic space also significantly influences the waterfront experience. The relatively lower weight assigned to water body interactivity suggests that residents currently prioritize accessibility and the basic environment of water-adjacent areas over direct engagement with the water. Therefore, enhancing hydrophilicity requires optimizing the layout and accessibility of hydrophilic facilities. Furthermore, significant potential exists for improving water body interactivity.

## Discussion

### Place symbiosis in action: an empirically grounded model

Our macro-level GIS analysis identified it as a prominent nighttime activity “hotspot” (Fig. 5), a pattern that remained unexplained at that scale. However, our micro-level behavioral mapping (Sect. 3.2.2.1) revealed the underlying symbiotic process: residents, driven by social and cultural needs, adaptively redefined the open space for evening group dancing. This interaction perfectly exemplifies “place symbiosis”: the residents’ activities (the “symbiotic” component) actively shape the place’s identity and function, while the spatial affordances of the square—its openness, location, and accessibility — (the “biotic” or environmental component) enable and sustain this activity. This dynamic feedback loop, where people and place continuously co-create each other, explains the square’s vitality far more powerfully than a simple analysis of its physical design. This empirically grounded model of “place symbiosis” thus moves beyond generic interaction frameworks, offering a more nuanced lens to understand and foster the endogenous vitality of urban public spaces.

### Renewal of public space surrounding lakes: from “Space Creation” to “Place Symbiosis”

The renewal of public spaces surrounding lakes should move beyond mere physical optimization towards “place reshaping,” aiming to stimulate vitality, strengthen human-place emotional connections, and foster a transition from generic “space” to meaningful “place.” Building on the preceding analysis, renewal strategies should transcend a purely functionalist paradigm to deeply consider residents’ diverse experiences. Accordingly, we propose the following renewal approaches.

#### Upholding the “Safety and Resilience” principle: targeted interventions based on user perception

Our empirical findings pinpointed critical safety deficiencies as the primary leverage point for renewal, with the AHP analysis identifying “Lighting Conditions” as the most critical safety sub-factor (Table 5) and subjective evaluations revealing users’ strong dissatisfaction with it (Fig. 7). Responding to these data-driven insights, our primary strategy is to enhance perceived safety through targeted interventions rather than merely adding facilities. This includes: (1) deploying a smart lighting system that dynamically adapts to pedestrian flow, with focused, warm-colored lighting in critical areas like watersides and steps to boost perceived safety—a practice supported by established research linking lighting quality to nighttime space use^16,17^; and (2) establishing rapid response mechanisms, including emergency call points and AEDs, alongside reinforcing waterside protection^18^. These measures align with the core principles of Crime Prevention Through Environmental Design (CPTED)^19^, which emphasizes enhancing territoriality and natural surveillance to reduce both actual crime and the fear of it, thus laying the groundwork for “safety-activated vitality.”

#### Pursuing a “Seamless Connection” experience: prioritizing efficiency based on resident values

Directly responding to the primacy of “Travel Time Cost”—identified in our AHP analysis (Table 5) as the most crucial accessibility factor—our strategy shifts towards minimizing travel friction. We propose a multi-dimensional connection network that: (1) optimizes the public transport network to reduce arrival times by adding bus stops, refining routes, and ensuring adequate parking; (2) champions a “slow mobility priority” through high-quality, safe pedestrian and cycling systems^20,21^ that integrate “fun paths” and “rest stations”; and (3) enhances information accessibility via smart wayfinding platforms^22^. In doing so, this approach operationalizes the core principles of the “15-Minute City” concept^23^, which advocates for an urban structure where residents can access most of their daily needs within a short walk or bike ride, thereby enhancing convenience and promoting sustainable urban health.

#### Adopting an “Ecological Integration” path: responding to high resident expectations for nature

The dual findings that “Green Coverage Rate” is the top-weighted comfort factor (Table 5) and resident satisfaction with nature is high (Fig. 7) create a strong, evidence-based mandate for ecological integration. We therefore propose an “ecological integration” path aimed at: (1) increasing high-quality green coverage by optimizing plant configurations and constructing continuous “ecological corridors”^24^; (2) creating comfortable microclimates by introducing concepts like “healing landscapes”^25–28^ and “sensory gardens”^29,30^; and (3) integrating Nature-based Solutions (NbS)^31^, such as rain gardens and bioswales, to enrich the waterside experience and enhance ecological resilience. This holistic strategy is a direct application of Biophilic Design principles^32^, a theory demonstrating that strengthening the human-nature connection in urban environments significantly enhances well-being, reduces stress, and fosters environmental stewardship.

#### Creating “Narrative” space: from Spatial heterogeneity to place identity

The significant spatial heterogeneity of activities revealed by our GIS analysis (Fig. 5), which shows distinct functional nodes and hotspots, directly informs our strategy to move beyond a monolithic renewal approach. Instead, the strategy should aim to amplify and enrich these existing spatial characters, elevating them from mere functional zones to “narrative places” with unique identities and meanings. We propose creating “place stories” by extracting and weaving the unique spat-temporal imprints of Qingshan Lake itself. This could include its ancient water conservancy legends, its modern history as a city park, or the characteristic water-based activities formed by contemporary citizens (e.g., dragon boat races, winter swimming clubs), reflecting unique local activity spaces. This requires operationalizing Norberg-Schulz’s concept of “Genius Loci”^33^: identifying the unique spirit of each area and integrating it into landscape, architectural, and signage design. For instance, the water area where dragon boat activities congregate could be themed as a “Dragon Boat Culture Stage,” while quiet ecological wetlands could be shaped into a “Migratory Bird Sanctuary Trail,” transforming the entire lakeside into an engaging network of unique, lake-related, story-driven places.

#### Shaping “Affordance” space: from observed adaptability to empowered Co-Creation

The strategy to shape “affordance” space stems directly from our behavioral mapping findings (Sect. 3.2.2.1), which captured numerous instances of residents creatively adapting and “redefining” public spaces. This observed user creativity—turning lawns into picnic spots or paved areas into temporary markets or dance floors—reveals a crucial insight: enduring vitality stems from empowering bottom-up co-creation, not from imposing top-down functions. Therefore, the ultimate renewal strategy is to shape the space’s “affordance”. This requires providing simple, durable, and versatile “substrates” (e.g., open lawns, flat platforms, structurally simple pavilions)^34^ with multiple potential uses, rather than rigidly programmed, single-function areas. This approach is a direct application of Gibson’s Theory of Affordances^35^ to urban design, a theory positing that the environment offers possibilities for action. By offering a flexible “scaffolding” for community co-creation rather than a limiting “finished product”, the design fosters an organic evolution of place functions. This aligns with contemporary scholarship on the “urban commons”, which argues for enabling residents to actively shape their shared environments and thereby cultivate an endogenous, vibrant, and resilient spirit of place^36^.

## Conclusion

This study makes a dual contribution to urban waterfront research. Methodologically, it pioneers and validates a multi-scale analytical framework that synergistically integrates macro-level GIS spatial analysis with micro-level insights from Behavior Setting Theory. This paradigm offers a reproducible and robust approach for investigating complex human-environment interactions in public spaces. Theoretically, it proposes and empirically grounds the concept of “place symbiosis,” moving beyond static place-making to articulate a dynamic, co-evolutionary process between residents and their environment.

Situated in the context of urban renewal, our case study of Nanchang’s Qingshan Lake applied this framework to reveal nuanced spatio-temporal patterns of space utilization, diverse activity preferences, and the mechanisms of user-space interaction. Building on these findings, the proposed micro-renewal strategy, centered on “place symbiosis,” transcends traditional “space creation” to champion quality improvement and vitality regeneration. Limitations include potential constraints on sample representativeness due to research duration and data acquisition methods, and the need for deeper exploration of resident behavioral motivations. Additionally, while kernel density analysis effectively identifies activity hotspots, it does not explain the causal factors driving their formation. Future research could integrate spatial configuration analysis (e.g., Space Syntax) or environmental variable overlays to provide a more comprehensive understanding of the underlying spatial drivers. Furthermore, we acknowledge that the use of a three-point Likert scale, while simplifying the survey for participants, may limit the granular analysis of satisfaction levels. Future studies could employ a five- or seven-point scale to capture more nuanced perceptions. Future research should also adopt a stronger people-oriented approach that focuses on the differentiated needs of diverse groups and social equity^37–39^. Integrating multi-source data, intelligent technologies (e.g., big data, VR/AR), and multidisciplinary perspectives (e.g., sociology, psychology, behavioral geography) will enable a more precise understanding of complex human-environment interactions. Continued theoretical innovation and practical exploration will be crucial to driving enhancements in resident well-being, strengthening community cohesion, and contributing to sustainable urban development, fostering harmonious coexistence between people, the city, and the lake environment.

## Data Availability

The datasets generated during and/or analysed during the current study are available from the corresponding author on reasonable request.
